# Lumbar Subarachnoid-Peritoneal Shunting Deteriorates Superficial Siderosis Associated with a Dural Defect

**DOI:** 10.7759/cureus.54651

**Published:** 2024-02-21

**Authors:** Narutada Ando, Yusuke Nakazawa, Takeshi Miyata, Takenori Ogura, Wataru Shiraishi, Taketo Hatano

**Affiliations:** 1 Neurosurgery, Kokura Memorial Hospital, Kitakyushu, JPN; 2 Neurology, Kokura Memorial Hospital, Kitakyushu, JPN; 3 Internal Medicine, Shiraishi Internal Medicine Clinic, Nōgata, JPN

**Keywords:** superficial siderosis, lumbar subarachnoid-peritoneal shunting, hemosiderin, cervical surgery, cerebrospinal pressure

## Abstract

Superficial siderosis is a disease in which hemosiderin is deposited under the leptomeninges and subpial layers of hindbrain structures, e.g., the cerebellum, brainstem, and eighth cranial nerve. The main symptoms of superficial siderosis are cerebellar ataxia, hearing loss, cognitive decline, and myelopathy. The activities of daily living of patients with superficial siderosis are severely impaired due to the progressive symptoms. Here, we report a patient with superficial siderosis whose symptoms deteriorated after lumbar subarachnoid-peritoneal (L-P) shunt surgery. She received L-P shunt surgery based on the diagnosis of idiopathic normal pressure hydrocephalus at another hospital. The patient had a history of cervical surgery, and a dural defect was identified at the C4-5 level by a detailed magnetic resonance imaging study. We hypothesized that the L-P shunt reduced cerebrospinal pressure and increased bleeding from the fragile vessels in the dural defect, which might have increased hemosiderin deposition.

## Introduction

Superficial siderosis is a rare disorder characterized by hemosiderin deposition in the leptomeninges and subpial layers [1]. Recurrent or continuous bleeding into the cerebrospinal fluid (CSF) has been implicated as a cause of superficial siderosis [2], and is reportedly found in more than half of all cases [3]. Apart from idiopathic superficial siderosis, iatrogenic superficial siderosis has been reported following hemispherectomy, spinal decompression surgery, posterior fossa surgery, and ventriculoperitoneal (V-P) shunt surgery [4]. 

Here, we report a patient with superficial siderosis whose symptoms deteriorated after lumbar subarachnoid-peritoneal (L-P) shunt surgery. The patient’s symptoms of ataxia, hearing loss, and dementia accelerated after L-P shunt surgery. She had a history of spinal surgery, and we identified a dural defect associated with this surgery using constructive interference in steady state (CISS) magnetic resonance imaging (MRI). We hypothesized that the L-P shunt reduced CSF pressure and increased bleeding from the postoperative dural defect, and eventually, it worsened her superficial siderosis symptoms.

## Case presentation

An 80-year-old Japanese woman with a two-year history of gait instability, hearing loss, and cognitive dysfunction was admitted to our department. She had a history of anterior cervical discectomy and fusion at 50 years of age and cervical laminectomy at 74 years of age. At the age of 78 years, she underwent L-P shunt surgery in another hospital based on the diagnosis of normal pressure hydrocephalus. However, she showed no amelioration of gait instability or cognitive impairment (Mini-Mental State Examination score: from 18/30 to 17/30 after a month). After L-P shunt surgery, her gait instability and cognitive dysfunction worsened, and hearing loss appeared. The patient was able to walk before the surgery, but after the surgery, she was unable to walk without a walker.

**Figure 1:**
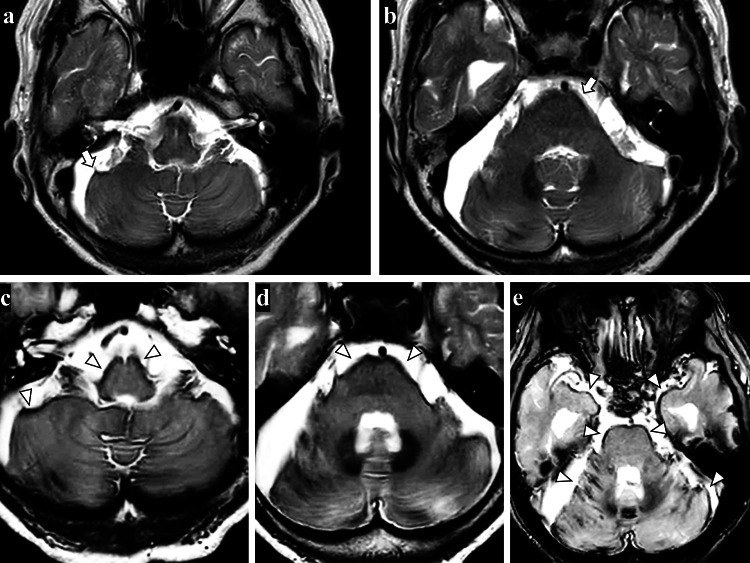
Pre- and postoperative brain MRI of the lumbar subarachnoid-peritoneal (L-P) shunt at the age of 78 years (a, b) and 80 years (c, d). (a, b) Preoperative axial T2-weighted MRI showing hemosiderin deposits on the surface of the brainstem and cerebellum (arrows); (c, d) At two years after L-P shunt surgery, T2-weighted MRI showing the increased deposition of hemosiderin (arrowhead); (e) T2*-weighted MRI showing numerous hemosiderin deposition (arrowheads).

At the age of 80 years, she fell, bruised her head, and visited our hospital. Due to her difficulty in moving, she was admitted to our department. On admission, neurological examination revealed ataxic and spastic gait disturbance, cerebellar dysarthria, sensory hearing loss, and cognitive dysfunction with a score of 12/30 on the Mini-Mental State Examination. Her deep tendon reflexes were normal in the upper extremities, but the lower extremities showed hyperreflexia. Babinski’s sign was positive bilaterally. CSF sampling showed xanthochromia and mild CSF protein elevation. MRI of the brain showed diffuse hemosiderin deposits presenting as a T2-weighted hypointense layer on the surface of the cerebellum and brainstem, which had worsened compared to brain MRI at the time of L-P shunt surgery (Figure 1). There were no findings of diffuse dural thickening or sagging of the chiasm.

**Figure 2:**
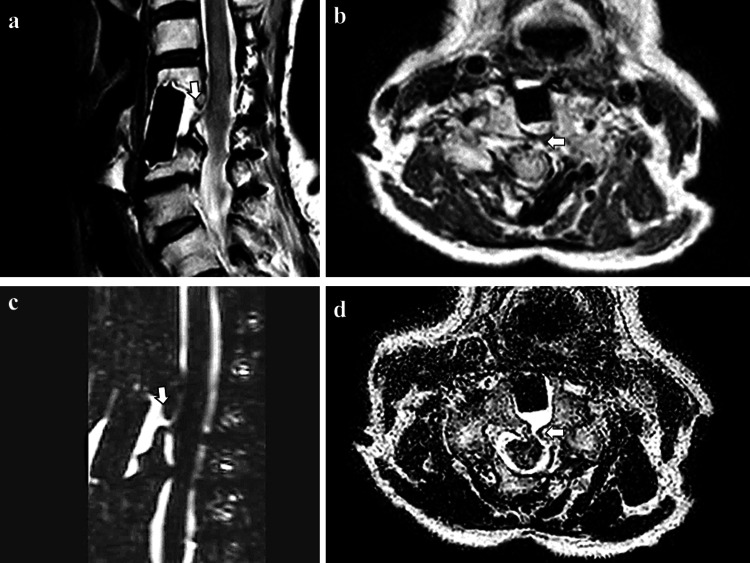
Cervical MRI (a) Sagittal T2-weighted cervical MRI and (b) axial T2-weighted cervical MRI showing a dural defect at the cervical level, which was a postoperative lesion of cervical discectomy; (c) Heavily T2-weighted cervical MRI showing the inflow of water into the dural fistula to the surgical discectomy and fusion lesion; (d) Axial constructive interference in steady state spinal MRI showing the dural defect clearer than T2-weighted MRI (b).

MRI myelography revealed evidence of a dural defect at the C4-5 level (Figure 2), which was more apparent on CISS MRI and heavily T2-weighted MRI.

We assumed that the dural defect had occurred during cervical spine surgery at age 50 years. As the symptoms worsened rapidly after L-P shunt surgery, it was considered that the L-P shunt had increased the bleeding from the fragile vessels in the dural defect, possibly due to shunt-induced low CSF pressure. As a treatment, we tied off the shunt valve and started oral chelating and hemostatic agents. After treatment, she showed no symptom progression. We also suggested dural repairment surgery to her, but the patient decided against this procedure.

## Discussion

This was a case of superficial siderosis deterioration after L-P shunt surgery. The patient had a history of cervical surgery at 50 and 74 years of age. Her brain MRI findings showed hemosiderin deposition, which was not severe at the age of 78 years but had worsened at two years after L-P shunt surgery.

Superficial siderosis is caused by continuous or recurrent bleeding into the subarachnoid space, resulting in the deposition of hemosiderin in the leptomeninges and subpial layers of hindbrain structures, especially the cerebellum, brainstem, and spinal cord [4,5]. Superficial siderosis is characterized by hearing loss (95%), cerebellar ataxia (88%), and pyramidal signs (76%) [3]. Dementia is less common, but cognitive, social, and emotional functions are reportedly impaired in patients with superficial siderosis [5]. Regardless of the location of the bleeding source, superficial siderosis predominantly affects the cerebellum, brainstem, and eighth cranial nerve. Animal studies suggest that ferritin-repressor protein-immunoreactive Bergmann glia of the cerebellum have a role in converting heme to ferritin and finally, to hemosiderin [2]. Blood pooling in the posterior fossa is also considered to predispose to this regional predominance [6]. The selective vulnerability of the eighth cranial nerve is probably due to the long glial segment that predisposes it to iron deposition [7].

Treatment of the bleeding source is the first choice approach for superficial siderosis. For this reason, it is crucial to identify the bleeding source. Still, as mentioned above, hemosiderin tends to be deposited in the cerebellum, brainstem, and eighth cranial nerve, so the hemosiderin deposition site(s) and bleeding source do not always match. Therefore, it is necessary to perform imaging examinations of the head and whole spinal cord to identify the source of bleeding. In neuroimaging findings, CISS MRI was reported to be useful for detecting dural defects in patients with superficial siderosis [8,9]. The current case also showed a more apparent dural defect on CISS MRI and heavily T2-weighted MRI than on normal T2-weighted MRI. Kumar et al. pointed out that the frequent association of fluid-filled cavities with superficial siderosis suggests that the associated dural defect may be the bleeding source. Indeed, our case showed such cavities on cervical CISS MRI [10].

Regarding the relationship between the shunt and superficial siderosis, there are some reports about shunt-associated superficial siderosis [1,4,11]. Kumar et al. presented the cases of two patients who developed superficial siderosis after shunt placement, but the clinical details were not described [10]. Satow et al. reported a case of V-P shunt complication in which a minor intraventricular hemorrhage was confirmed one day after V-P shunt surgery [4]. They also suggested that an L-P shunt was a safer method for patients with superficial siderosis. McCarron et al. (2003) described a patient who developed superficial siderosis over a number of years after multiple shunt revisions (ventriculoatrial and V-P shunts) following posterior fossa surgery for Chiari malformation [11]. McCarron et al. (2004) also reported that shunt surgery for superficial siderosis resulted in improved CSF red blood cell count, but no clinical recovery [12]. As indicated in these reports, CSF hypotension and superficial siderosis have a close relationship [10]. 

**Figure 3:**
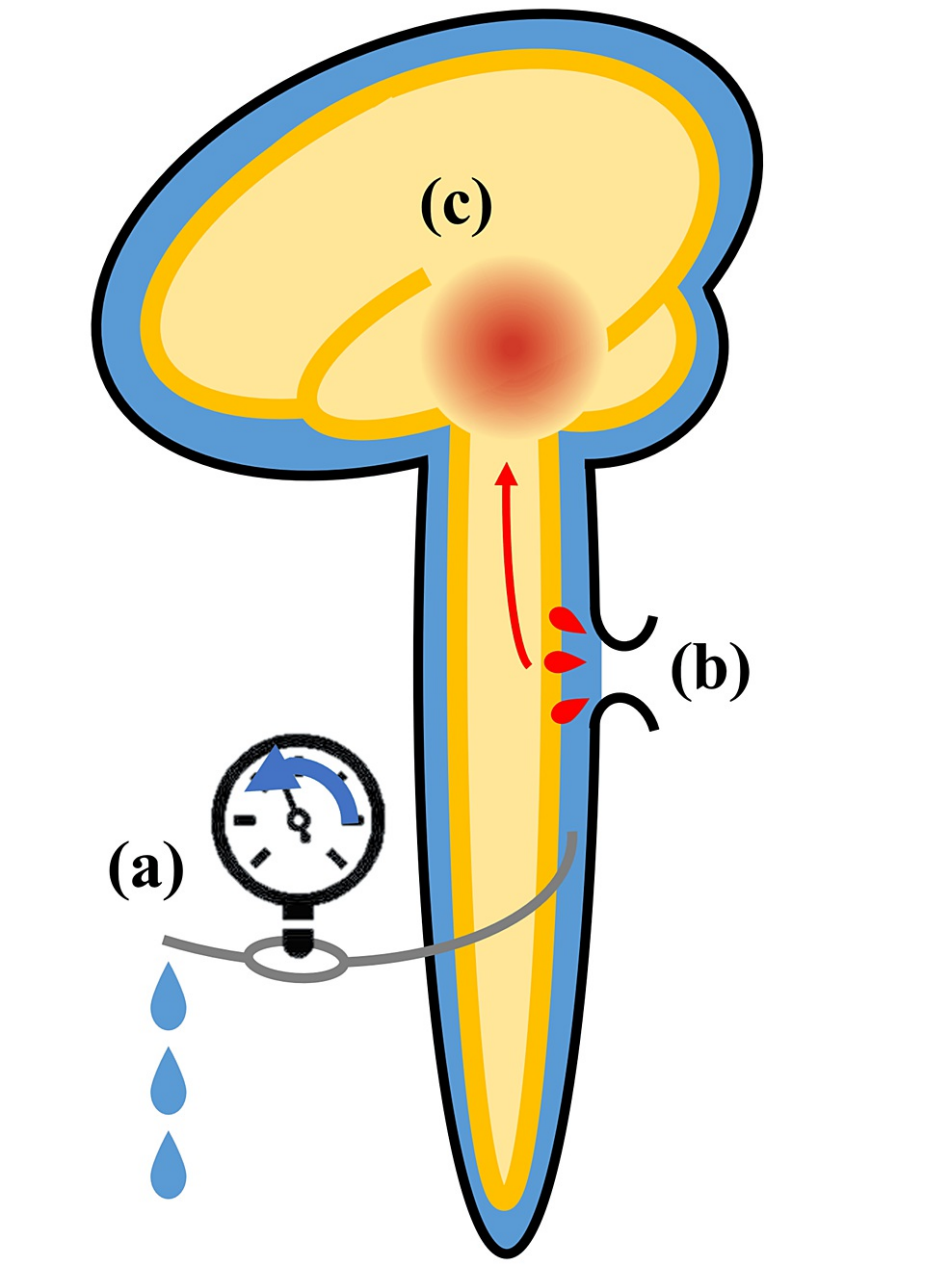
A schematic of the pathophysiological hypothesis in the current case (a) First, the lumbar-subarachnoid peritoneal shunt reduced cerebrospinal fluid (CSF) pressure; (b) Second, bleeding from the fragile vessels in the dural defect was increased due to the reduction of CSF pressure; (c) Third, increased bleeding into the CSF led to the increased deposition of hemosiderin and deterioration of superficial siderosis. Image credit: Authors

In the current report, the dural defect might have occurred following cervical spine surgery at the age of 50 or 74 years; however, the patient's clinical symptoms of superficial siderosis deteriorated after L-P shunt surgery. We made the hypothesis that the L-P shunt procedure decreased CSF pressure, resulting in an increase of bleeding from the fragile vessels in the dural defect. This increased bleeding was considered to have accelerated the deposition of hemosiderin, leading to the worsening of superficial siderosis symptoms such as hearing loss, ataxia, and cognitive decline (Figure 3). We considered this hypothesis because of the rapid deterioration after the L-P shunt surgery.

Since the relationship between shunt surgery and bleeding from fragile vessels in the dural defect is a hypothesis at present, it is essential to accumulate similar cases.

## Conclusions

For patients at risk of superficial siderosis, such as those with a dural defect or post-cervical spinal surgery, medical procedures that reduce CSF pressure, such as L-P and V-P shunts, may exacerbate the symptoms of superficial siderosis by increasing bleeding into the CSF. Clinicians need to be aware that shunt surgery can be a deteriorating factor for superficial siderosis. Furthermore, CISS MRI may help detect the dural deficit, which can be the source of bleeding in intractable cases of superficial siderosis.

## References

[1] Kumar N, Cohen-Gadol AA, Wright RA, Miller GM, Piepgras DG, Ahlskog JE (2006). Superficial siderosis. Neurology.

[2] Koeppen AH, Dickson AC, Chu RC, Thach RE (1993). The pathogenesis of superficial siderosis of the central nervous system. Ann Neurol.

[3] Fearnley JM, Stevens JM, Rudge P (1995). Superficial siderosis of the central nervous system. Brain.

[4] Satow T, Yamada S, Yagi M, Saiki M (2010). Superficial siderosis of the central nervous system after ventriculoperitoneal shunt. J Neurosurg.

[5] van Harskamp NJ, Rudge P, Cipolotti L (2005). Cognitive and social impairments in patients with superficial siderosis. Brain.

[6] Bracchi M, Savoiardo M, Triulzi F (1993). Superficial siderosis of the CNS: MR diagnosis and clinical findings. AJNR Am J Neuroradiol.

[7] Revesz T, Earl CJ, Barnard RO (1988). Superficial siderosis of the central nervous system presenting with longstanding deafness. J R Soc Med.

[8] Hosokawa M, Murata KY, Hironishi M, Koh J, Nishioka K, Nakao N, Ito H (2018). Superficial siderosis associated with duplicated dura mater detected by CISS reverse MRI. J Neurol Sci.

[9] Sakoda A, Yamashita KI, Hayashida M, Iwamoto Y, Yamasaki R, Kira JI (2017). A case of superficial siderosis ameliorated after closure of dural deficit detected by MRI-CISS (constructive interference in steady state) imaging [Article in Japanese]. Rinsho Shinkeigaku.

[10] Kumar N, McKeon A, Rabinstein AA, Kalina P, Ahlskog JE, Mokri B (2007). Superficial siderosis and csf hypovolemia: the defect (dural) in the link. Neurology.

[11] McCarron MO, Flynn PA, Owens C, Wallace I, Mirakhur M, Gibson JM, Patterson VH (2003). Superficial siderosis of the central nervous system many years after neurosurgical procedures. J Neurol Neurosurg Psychiatry.

[12] McCarron MO, Patterson VH (2004). Effects of shunting CSF in superficial siderosis of the CNS. Neurology.

